# The weekend effect for stroke patients admitted to intensive care: A retrospective cohort analysis

**DOI:** 10.1371/journal.pone.0234521

**Published:** 2020-06-10

**Authors:** William Greig Mitchell, Rohit Pande, Tom Edward Robinson, Gabriel Davis Jones, Isabella Hou, Leo Anthony Celi

**Affiliations:** 1 Harvard T.H. Chan School of Public Health, Boston, MA, United States of America; 2 Whitireia Community Polytechnic, Porirua, New Zealand; 3 University of Auckland, Auckland, New Zealand; 4 Oxford University, Oxford, United Kingdom; 5 Southern Cross Health Society, Auckland, New Zealand; 6 Beth Israel Deaconess Medical Centre, Harvard Medical School, Massachusetts Institute of Technology, Cambridge, MA, United States of America; University of Ioannina School of Medicine, GREECE

## Abstract

**Objectives:**

To examine the effect of weekend admission on short and long-term morbidity and mortality, for patients admitted to intensive care after suffering a cerebrovascular accident (stroke).

**Design, setting, and participants:**

A hospital-wide, retrospective cohort study of 3,729 adult stroke patients admitted to the Beth Israel Deaconess Medical Centre (BIDMC) intensive care unit (ICU) between 2001 and 2012, using the Medical Information Mart for Intensive Care III (MIMIC-III) database.

**Primary outcome measures:**

Primary outcome measures were ICU length-of-stay and mortality, hospital length-of-stay and mortality, proportions of patients discharged home after admission, and 6-month mortality.

**Results:**

Overall, 23% of BIDMC ICU stroke admissions occurred over the weekend. Those admitted over the weekend were likelier to have suffered haemorrhagic stroke than those admitted during the week (60.6% vs 47.9%). Those admitted on the weekend were younger, and likelier to be male and unmarried, with similar ethnic representation. The OASIS severity of illness (32.5 vs. 32) and lowest day-one GCS (12.6 vs. 12.9) were similar between groups. Unadjusted ICU-mortality was significantly higher for patients admitted over the weekend (OR 1.32, CI 1.08–1.61), but when adjusted for type of stroke, became non-significant (OR 1.17, CI 0.95–1.44). In-hospital mortality was significantly higher for patients admitted to ICU over the weekend in both unadjusted (OR 1.45, CI 1.22–1.73) and adjusted (OR 1.31, CI 1.09–1.58) analyses. There was no significant difference in ICU or hospital length of stay. While patients admitted on the weekend appeared less likely to be discharged back to home and more at risk of 6-month mortality compared to weekday admissions, results were non-significant.

**Conclusions:**

The effect of weekend ICU-admission for stroke patients appears to be significant for in-hospital mortality. There were no significant differences in adjusted ICU-mortality, ICU or hospital length-of-stay, or longer-term morbidity and mortality measures.

## Introduction

The effect of weekend admission on increased hospital mortality is well described [1–5], but with conflicting consistency across hospitals and specialties [6–10]. In-hospital factors such as lower overnight staffing [11, 12], less experienced physicians [13], fewer ward rounds [12], delayed assessment and management [12]–and pre-hospital factors such as sicker patient populations [14] or higher acuity of illness [15], are all posited as potential contributors toward the phenomenon of higher mortality for those admitted to hospital over the weekend (henceforth referred to as the ‘weekend effect’).

Because the incidence of critical illness does not change over the weekend [16], and patients admitted to intensive care (ICU) require timely, intensive, 24-hour care [12], ICUs have increased levels of weekend staffing and experience compared to the rest of the hospital [10], in an effort to minimize the weekend mortality discrepancy. Despite this, there is a demonstrably higher mortality risk for patients admitted to ICU on a weekend [12, 14, 16–18]. A thorough investigation of the weekend effect for stroke patients admitted to ICU, assessing not only immediate mortality, but longer-term mortality and morbidity outcomes, is yet to be described.

While the weekend effect for stroke patients admitted to a hospital ward has been shown to be minimal [2, 7, 19], pre-hospital factors influencing mortality (like stroke severity) may uniquely apply to the population of patients admitted to an ICU rather than a ward, over the weekend. Stroke patients admitted to an ICU have likely suffered more severe strokes than those admitted to a ward, and are likely at greater risk of sudden deterioration; requiring particularly close monitoring for potentially immediate intervention. Because of this, weekend staffing and experience differentials in the ICU may affect outcomes of critically unwell stroke patients (where timely assessment and management is particularly important [7]) differently to how it affects stroke patients admitted to a general ward. Finally, extending outcome measurements to determine level of disability at hospital discharge, and long-term mortality, may outline whether the implications of weekend admission to an ICU for stroke patients extend beyond acute hospital outcomes.

Our study describes short and long-term morbidity and mortality outcomes of an adult population suffering stroke, admitted to an academic center ICU in Boston, Massachusetts, USA, with the intention of eliciting so-far undescribed short and long-term admission outcomes for a critically unwell patient population, admitted on a weekend.

## Methods

This was a hospital-wide, retrospective cohort study using the Medical Information Mart for Intensive Care III (MIMIC-III) database from the Beth Israel Deaconess Medical Centre (BIDMC) [20]. MIMIC-III is a large, publicly-available database of de-identified health-related data, collected prospectively for over 40,000 patients who were admitted to the BIDMC ICU. The database includes basic demographic information, admission details (laboratory findings and investigations, medications, interventions and outcomes) and encompasses a diverse, large population of critically ill patients in the United States. The data in this study was limited to patients aged 16–89 years of age. All data is de-identified in accordance with the Health Insurance Portability and Accountability Act (HIPAA) standards, including removal of names, phone numbers, and addresses. Date-shifting was utilised to preserve de-identification, however time of the day and day of the week are preserved in the MIMIC III database [20]. The institutional review boards of the Massachusetts Institute of Technology (No. 0403000206) and BIDMC (2001-P-001699/14) both approved the use of the database for research.

Stroke was defined using ICD-9 codes recorded in the MIMIC III database. We defined stroke with ICU admission diagnosis classified as ICD-9 code 430 (subarachnoid haemorrhage), 431 (intracerebral haemorrhage), 432 (other and unspecified intracranial haemorrhage), 433 (occlusion and stenosis of precerebral arteries), or 434 (occlusion of cerebral arteries) [21]. Stroke was further subclassified into either haemorrhagic (ICD 430, 432, 432) or ischemic (ICD 433, 434) stroke. Weekend ICU admission was defined as any admission during the 48-hour period between midnight at the beginning of Saturday, and midnight at the end of Sunday.

The MIMIC III database records acute severity of illness using the Oxford Acute Severity of Illness Score (OASIS); a simplified illness severity score developed using machine learning [22]. It has a discrimination, calibration and prediction of equivalent accuracy to more complex models like APACHE IV [22], and is used in our analysis as an indicator of illness severity (Table 1).

**Table 1:** Baseline demographics for weekend versus weekday stroke admissions to intensive care.

	Group 1	Group 2
	Weekend admission	Weekday admission
**No. of stroke admissions (n, %)**	858 (23.0)	2,871 (77.0)
**Haemorrhagic stroke**	520 (60.6)	1,374 (47.9)
- **Ischemic stroke**	338 (39.4)	1,497 (52.1)
**Age (mean, SD)**	65.4 (15.6)	67.2 (14.5)
**Female (n, %)**	380 (44.3)	1,332 (46.4)
**Married (n, %)**	441 (51.4)	1,503 (52.3)
**Ethnicity (n, %)**		
- **Black**	65 (7.6)	196 (6.8)
- **White**	611 (71.2)	2,094 (72.9)
- **Other**^**a**^	106 (12.3)	347 (12.1)
- **Unknown**	76 (8.8)	234 (8.1)
**Insurance status**^**c**^ **(n, %)**		
- **Medicare/Medicaid**	528 (61.5)	1,879 (65.4)
**Admission type**^**d**^ **(n, %)**		
- **Emergency**	847 (98.7)	2,591 (90.2)
**OASIS**^**b**^ **severity of illness (mean, SD)**	32.5 (8.7)	32 (8.8)
**Lowest GCS (D1) (mean, SD)**	12.6 (3.4)	12.9 (3.3)

Key

a: Other = Combination of Hispanic/Latino, Asian, or other

b: OASIS: a modified severity of illness score, with similar discrimination and predictive capacity as the APACHE severity of illness scores (18)

c: Insurance = Either Medicare/Medicaid or private/self-funded

d: Admission type = Either emergency (including urgent or emergency) or non-emergency

Only the first index admission to ICU for each patient was included in the analysis, to avoid multiple analyses for patients re-admitted to the intensive care for a presentation associated with their initial cerebrovascular accident.

Demographic and outcome data is presented as either counts and percentages (for categorical data), or means and standard deviations (for continuous data). Ethnicity is divided into four primary categories (“Other” inclusive of Hispanic, Latino, Asian and other patients) to preserve statistical power, insurance status as either government (Medicare, Medicaid) or non-government, and ICU admission type as either emergency (urgent or emergency admission to ICU) or elective.

The ICU and hospital length of stay (LOS) was calculated only for those who survived the index ICU or hospital admission. The discharge home and 6-month mortality outcomes were calculated only for those who survived the index hospital admission, in order to represent the difference in the level of disability at the end of admission between groups, and the longer-term mortality of those who survived initial hospital admission, respectively.

Differences in demographics and outcome data were calculated using Student’s t-test, Chi-squared test, unadjusted univariable logistic regression and adjusted multivariable logistic regression for significant confounding variables (adjusted for age, gender, and admission type). A p-value of less than 0.05 was considered statistically significant, and odds ratios are presented with 95% confidence intervals. Missing data was present for <0.1% of all patient information, and any patient with missing data was excluded from that particular analysis. All analyses were performed using Python 3.6.9.

This project is an output of Hack Aotearoa 2020, a health datathon organized by the University of Auckland and MIT Critical Data. This research received no specific grant from any funding agency in the public, commercial or not-for-profit sector. There are no competing interests to declare by any of the authors.

## Results

A total of 3,729 patients aged 16–89 years old were admitted to the Beth Israel Deaconess Medical Centre (BIDMC) ICU between 2001 and 2013 for an acute cerebrovascular event (stroke). Missing data was present for <0.1% of participants during this period, and patients with missing data were excluded from any analysis for that domain.

### Baseline characteristics

Overall, 23% of BIDMC ICU stroke admissions occurred over the weekend, and 77% occurred during the week (Table 1). Stroke patients admitted to ICU over the weekend were more likely to have suffered haemorrhagic strokes vs ischemic strokes (60.6% vs 39.4%), with more similar distribution of haemorrhagic vs ischemic stroke for those admitted during the week (47.9% vs 52.1%) (Table 1). Those admitted on the weekend were younger (65.4 vs. 67.2 years old), and more likely to be male (55.7% vs. 53.6%) and unmarried (48.6% vs. 47.7%). Ethnic subcategory representation was comparable between weekend and weekday ICU admission (Table 1), and those admitted to the ICU on the weekend were less likely to be publicly insured (61.5% vs. 65.4%) than those admitted on a weekday (Table 1). Those admitted on the weekend were more likely to be emergency admissions, compared to those admitted during the week (8.7% vs. 90.2%). The OASIS severity of illness score was similar for patients admitted to ICU on the weekend and the weekday (32.5 vs. 32), and the lowest day-one Glasgow Coma Scale (GCS) score was also similar between both groups (12.6 vs. 12.9) (Table 1).

### Intensive care outcomes

While ICU length of stay appeared similar for stroke patients admitted on the weekend compared to those admitted on a weekday (5.6 vs. 5.3 days), results were non-significant (Table 2). The ICU mortality rates were significantly higher for stroke patients admitted on the weekend compared to a weekday (18.8% vs. 14.9%, p = 0.006) (Table 2). Unadjusted odds of ICU mortality were significantly higher for stroke patients admitted to intensive care over the weekend (OR 1.32, CI 1.08–1.61, p = 0.006) (Table 3). When adjusting for age, gender, and ICU admission type, odds remained significantly higher, however when adjusting for type of stroke, the higher likelihood of ICU mortality for those admitted over the weekend became non-significant (OR 1.17, CI 0.95–1.44, p = 0.13) (Table 3).

**Table 2:** Overall immediate, short and long-term outcomes for stroke patients admitted to intensive care.

	Group 1 Weekend admission	Group 2 Weekday admission	P value
**Intensive care outcomes**			
**ICU mortality (n, %)**	161 (18.8)	427 (14.9)	0.006
**ICU LOS (days, SD)**	5.6 days (6.7)	5.3 days (7.1)	0.27
**Hospital outcomes**			
**Hospital mortality (n, %)**	228 (26.6)	573 (20.0)	<0.001
**Hospital LOS (days, SD)**	11.2 days (11.6)	11.3 days (11.6)	0.92
**Discharge and long-term outcomes**			
**Discharge to home**^**a**^ **(mean, SD)**	234 (37.1)	937 (40.1)	0.10
**6-month**^**b**^ **mortality**	90 (14.3)	292 (12.7)	0.30

Key

a: percentages of the total number of those who survived hospital admission, to mark (of those who survived) how many had lower level of disability at discharge, and were able to be discharged home

b: percentages of the total number of those who survived hospital admission, to mark (of those who survived) longer-term mortality outcomes

**Table 3:** Univariable and adjusted multivariable logistic regression for short and long-term outcomes.

	Odds Ratio^b^ (95% CI)	P value
**ICU mortality outcomes**		
- Unadjusted ICU mortality	1.32 (1.08–1.61)	0.006
- Adjusted^a^ ICU mortality	1.17 (0.95–1.44)	0.13
**Hospital mortality outcomes**		
- Unadjusted hospital mortality	1.45 (1.22–1.73)	<0.001
- Adjusted^a^ hospital mortality	1.31 (1.09–1.58)	0.004
**Discharge home outcomes**		
- Unadjusted discharge home	0.86 (0.72–1.03)	0.10
- Adjusted^a^ discharge home^c^	0.88 (0.72–1.06)	0.18
**6-month mortality outcomes**		
- Unadjusted 6-month mortality	1.14 (0.89–1.48)	0.30
- Adjusted^a^ 6-month mortality^d^	1.12 (0.86–1.46)	0.41

Key

a: Adjusted for: age, gender, admission type, and stroke type (reference: male, elective, hemorrhagic)

b: Reference group–weekday admission to intensive care

c: percentages of the total number of those who survived hospital admission, to mark (of those who survived) how many had lower level of disability at discharge, and were able to be discharged home

d: percentages of the total number of those who survived hospital admission, to mark (of those who survived) longer-term mortality outcomes

### Hospital outcomes

While hospital length of stay appeared similar for stroke patients admitted on the weekend compared to those admitted on a weekday (11.2 days vs. 11.3 days), results were non-significant (Table 2). The hospital mortality rate was significantly higher for stroke patients admitted to ICU over the weekend (26.6% vs. 20%, p<0.001) (Table 2). Unadjusted odds of hospital mortality showed those admitted on the weekend had 1.45-times the odds of death compared to those admitted during the week (CI 1.22–1.73, p<0.001) (Table 3). When adjusted for age, gender, ICU admission type and type of stroke, the odds of in-hospital death for those admitted to ICU over the weekend remained significantly higher (OR 1.31, CI 1.09–1.58, p = 0.004) (Table 3).

### Discharge outcomes

The likelihood of being discharged to home for those admitted to ICU on the weekend compared to those admitted on a weekday was similar (37.1% vs. 40.1%, p = 0.10) (Table 2). Unadjusted odds of being discharged home for those admitted on the weekend compared to a weekday was similarly non-significant (OR 0.86, CI 0.72–1.03, p = 0.10) (Table 3), and remained non-significant when adjusting for age, gender, admission type, and type of stroke (OR 0.88, CI 0.72–1.06, p = 0.18) (Table 3).

### Long-term mortality outcomes

Stroke patients admitted to the ICU on the weekend appeared to have no significantly higher risk of 6-month mortality (14.3% vs. 12.7%, p = 0.30) (Table 2). Unadjusted odds of 6-month mortality for those admitted on a weekend compared to a weekday were non-significant (OR 1.14, CI 0.89–1.48, p = 0.30) (Table 3). When adjusted for age, gender, admission type and type of stroke, the odds of longer-term mortality for those admitted on the weekend compared to a weekday remained non-significant (OR 1.12, CI 0.86–1.46, p = 0.41) (Table 3).

## Discussion

The major findings of this study are an initially higher ICU mortality for stroke patients admitted over the weekend that becomes non-significant when adjusting for type of stroke, and a higher in-hospital mortality thereafter which remains significantly higher when adjusting for illness severity and type of stroke on admission. There was no significant difference in length of ICU and hospital admission between groups in this study involving more than 3,700 patients. While differences in the level of disability at discharge (denoted as those well enough to be discharged back home after their hospital admission) and longer-term outcomes (denoted as 6-month mortality of the hospital survivors) were non-significant, patients admitted on the weekend did appear to fare more poorly in both measures. This suggests the need for a larger study of the longer-term implications of weekend intensive care admission after a severe stroke as the effect size may not be big enough to detect given the sample size.

Although the weekend effect for patients admitted to intensive care has been previously reported as non-significant in older studies [6, 8, 10], a recent systematic-review and meta-analysis by Cavallazzi and colleagues [12] concluded that patients admitted to intensive care over the weekend were at significantly higher risk of both ICU and in-hospital mortality across multiple diagnoses [12]; partially consistent with our findings. This was posited to be due to in-hospital factors; lower levels of staffing and intensity of care over the weekend [12]. However, this analysis studied a general ICU population, rather than a sub-population for whom timely assessment and expert management may be particularly important, and the implications of a weekend admission more exaggerated. More recently, a systematic-review and meta-analysis by Galloway and colleagues found that admission to ICU over a weekend was also associated with a significant increased odds of death [16], also partially consistent with our findings; attributing in-hospital factors (an absence of on-site intensivists over the weekend compared to a weekday) toward the mortality differential. Of note, while on-site specialists are present in the BIDMC ICU during both weekdays and weekends, a significant proportion of specialists and nurses in the ICU over the weekend usually work in different departments (i.e. ambulatory) during the week; assisting weekend ICU staffing only where needed. There are also fewer junior medical staff working in the ICU over the weekend.

In addition to the in-hospital factors that may influence weekend mortality, pre-hospital factors like severity of illness have also been posited as influential. A 2019 systematic-review and meta-analysis by Chen and colleagues concluded that the significant increase in in-hospital weekend mortality was in part because patients admitted over the weekend were more severely ill [14]. In the present study, pre-hospital illness severity measures (OASIS, GCS) were similar between weekend and weekday admissions and did not influence mortality outcomes. However, those admitted over the weekend were considerably more likely to have suffered haemorrhagic strokes; considered more severe than ischemic strokes [23] and with previously reported higher rates of in-hospital mortality for those admitted over a weekend [15, 24]. Haemorrhagic strokes account for the vast minority of strokes [25], and the reason they were so disproportionately over-represented on weekend compared to weekday ICU admission in our cohort cannot be concluded by the present study. But when adjusting for type of stroke, higher odds of in-ICU mortality for those admitted over the weekend became non-significant; consistent with the notion that pre-hospital factors may play a particularly important role in the weekend mortality effect for those in ICU; in contrast to the suggested in-hospital contributors by Cavallazzi and colleagues and Galloway and colleagues (above).

Importantly, our study demonstrated higher in-hospital mortality for those admitted to ICU over the weekend even when accounting for pre-hospital factors; contrasting recent stroke-specific findings: Inoue and colleagues’ analysis of the weekend effect for stroke patients found no significant increased risk of in-hospital mortality for those admitted to a stroke-ICU over the weekend, after adjusting for severity of illness [7]. They studied ischemic stroke only, in an exclusively Japanese population, and with alternative acute illness severity and comorbidity measures (the modified Rankin Scale); which may in part explain the different findings of their study. A less recent 2010 study by Hoh and colleagues similarly described no significantly different adjusted in-hospital mortality for stroke patients admitted on the weekend [19] (similarly contrasting our findings). However, while their study size was significantly larger, they did not include or adjust for any measures of severity of illness or co-morbidity status (which may have considerably altered their findings), and similarly only studied an ischemic-stroke population. Neither study examined the implications of weekend admission on longer-term mortality patterns.

The effect of weekend admission to ICU for patients suffering severe stroke on in-hospital mortality appears significant for our study population, notably persisting even when adjusting for type of stroke (in contrast to previous studies [7, 14]). This outlines the potential effect of in-hospital factors on the ‘weekend mortality effect’, as alluded to by Zampieri and colleagues [11]. Further, the non-significant higher odds of in-ICU mortality for those admitted over the weekend demonstrates the potential influence of pre-hospital factors on ICU mortality for weekend admissions. The present findings underline the particular need for urgent review and management of patients who suffer severe stroke of any kind, where delay may substantially affect outcomes more-so than for other patient sub-populations. Lower numbers of on-site senior staff, lower nurse-to-patient ratios, fewer standardized protocols, and subsequent delays to expert management [11], may have more serious ramifications for severe stroke patients, underlying a sub-population of critically ill patients who need particular care when admitted over the weekend. Our findings are relevant for all healthcare professionals working both in hospital, or on-call from home, responsible for such an at-risk patient population.

### Strengths and limitations

This is the first study to analyse both the short-term and longer-term morbidity and mortality outcomes for stroke patients admitted to an intensive care unit over a weekend. Our database is large, inclusive of important information like severity of illness, and includes high-resolution patient information from a single center academic institution. Our analysis team comprised of clinicians, data scientists and statisticians, and the database used is publicly-available. This is congruent with an effort to move toward a more reproducible multidisciplinary, collaborative style of research.

As with all retrospective database research, there are limitations to this study. Information bias exists; stroke-specific markers of disability (i.e. the NIH Stroke Scale) were not recorded, so surrogate markers (discharged to home) used instead. And despite being a large dataset, this was only a single-center study, which limits generalizability

## Conclusion

In this large study of a diverse US population, the effect of weekend ICU admission for severe stroke patients appears to be significant for in-hospital mortality, irrespective of severity of illness or type of stroke. There were no significant differences in adjusted ICU mortality, or in ICU or hospital length of stay for those admitted on a weekend. While longer-term morbidity and mortality findings were non-significant, patients admitted over the weekend did appear to fare more poorly than those admitted on a weekday, which may warrant further exploration. Our findings are relevant to healthcare professionals working either in hospital or on-call from home, responsible for caring for such an at-risk patient population.
